# Characterization and Genetic Analysis of a Novel Light-Dependent Lesion Mimic Mutant, lm3, Showing Adult-Plant Resistance to Powdery Mildew in Common Wheat

**DOI:** 10.1371/journal.pone.0155358

**Published:** 2016-05-13

**Authors:** Fang Wang, Wenying Wu, Dongzhi Wang, Wenlong Yang, Jiazhu Sun, Dongcheng Liu, Aimin Zhang

**Affiliations:** 1 College of Agronomy/The Collaborative Innovation Center of Grain Crops in Henan, Henan Agricultural University, Zhengzhou, China; 2 State Key Laboratory of Plant Cell and Chromosome Engineering, Institute of Genetics and Developmental Biology, Chinese Academy of Sciences, Chaoyang District, Beijing, China; 3 University of the Chinese Academy of Sciences, Beijing, China; National Key Laboratory of Crop Genetic Improvement, CHINA

## Abstract

Lesion mimics (LMs) that exhibit spontaneous disease-like lesions in the absence of pathogen attack might confer enhanced plant disease resistance to a wide range of pathogens. The LM mutant, lm3 was derived from a single naturally mutated individual in the F_1_ population of a 3-1/Jing411 cross, backcrossed six times with 3–1 as the recurrent parent and subsequently self-pollinated twice. The leaves of young seedlings of the *lm3* mutant exhibited small, discrete white lesions under natural field conditions. The lesions first appeared at the leaf tips and subsequently expanded throughout the entire leaf blade to the leaf sheath. The lesions were initiated through light intensity and day length. Histochemical staining revealed that lesion formation might reflect programmed cell death (PCD) and abnormal accumulation of reactive oxygen species (ROS). The chlorophyll content in the mutant was significantly lower than that in wildtype, and the ratio of chlorophyll *a/b* was increased significantly in the mutant compared with wildtype, indicating that lm3 showed impairment of the biosynthesis or degradation of chlorophyll, and that Chlorophyll *b* was prone to damage during lesion formation. The *lm3* mutant exhibited enhanced resistance to wheat powdery mildew fungus (*Blumeria graminis* f. sp. *tritici*; *Bgt*) infection, which was consistent with the increased expression of seven pathogenesis-related (*PR*) and two *wheat chemically induced* (*WCI*) genes involved in the defense-related reaction. Genetic analysis showed that the mutation was controlled through a single partially dominant gene, which was closely linked to *Xbarc203* on chromosome 3BL; this gene was delimited to a 40 Mb region between *SSR3B450*.*37* and *SSR3B492*.*6* using a large derived segregating population and the available Chinese Spring chromosome 3B genome sequence. Taken together, our results provide information regarding the identification of a novel wheat LM gene, which will facilitate the additional fine-mapping and cloning of the gene to understand the mechanism underlying LM initiation and disease resistance in common wheat.

## Introduction

Lesion mimic mutants (LMMs) in plants spontaneously produce cell death or necrotic lesions in the absence of any pesticide, mechanical damage and pathogen infection [1]. The hypersensitive response (HR), localized cell death at sites of pathogen infection, is an innate immune response to protect plants from pathogen attacks, representing a form of programmed cell death (PCD) and conferring necrosis lesions to inhibit pathogen spread [2, 3]. Spontaneous cell death without the pathogen-induced HR response typically occurs in LMMs, which display necrotic lesions compared with plants infected with a pathogen. The necrotic spots and chlorotic leaves of LMMs result from the altered regulation of cell death processes, such as the HR and senescence or the perturbation of metabolic pathways, resulting in cell death [4]. During the HR process, generation of reactive oxygen species (ROS), accumulation of callose, production of phytoalexins and activation of the expression of pathogenesis-related (PR) genes generally occurs [5, 6]. Therefore, these lesion mimic (LM) genes might play a dual role in regulating cell death and activating plant defenses.

To reveal the function of LMs, numerous LMMs and their encoding genes have been identified from various plant species, such as *Arabidopsis*, rice, maize, barley and wheat [1, 7–14]. In rice, *spl7* (Spotted-leaf, *spl*) was the first isolated spotted-leaf gene, encoding a heat stress transcription factor (HSF) protein, and *spl7* lesions are induced in response to high temperature and ultraviolet solar irradiation [15]. Thereafter, various genes were shown to be involved or controlled in cell death and resistance responses, such as those encoding the E3 ubiquitin ligase protein (Spl11) [9], coproporphyrinogen Ⅲ oxidase (CPOX) protein [1, 16], splicing factor 3b subunit 3 [10, 11], and ATPase associated with various cellular activity type (AAA-type) proteins [17]. Early leaf senescence and lesion formation are observed in mutants for several genes, such as clathrin-associated adaptor protein [18], Mitogen-Activated Protein Kinase Kinase Kinase1 [19], and UDP-N-acetylglucosamine pyrophosphorylase 1 [20]. Moreover, several rice LMM genes have been fine-mapped, including *Lmes1* in an 88-kb region on chromosome 7 [21], *Spl30* delimited to a 94-kb region on chromosome 3 [22], and *spl2* and *spl6* on chromosomes 2 and 1, respectively [23, 24]. In *Arabidopsis*, a number of LM genes play an essential role in chloroplast development, resulting in small necrotic spots on plant leaves, such as chloroplast chaperonin 60β (*len1*) [25], membrane-remodeling GTPase (*fzl*) [26], and porphobilinogen deaminase (*rug1*) [27]. In addition, *lin2* encodes a CPOX protein, similar to *llm1* and *rlin1* in rice [28, 29], and several LM genes have been revealed to alter the expression of *PR* genes during the formation of spontaneous LMs [30–32]. In addition to the above mutated genes identified in rice and Arabidopsis, a few genes involved in necrotic lesion formation have also been characterized through overexpression or RNAi experiments in other species, including tomato [33], tobacco [34], and cotton [35, 36].

Because of the complexity of the wheat genome and incomplete sequence information, only a few LMMs have been identified or mapped in wheat [12–14, 37], the majority of which are invloved in pathogen resistance. The hypersensitive-like phenotype (HLP) mutant was obtained through a mutagenic treatment on the Argentine cultivar Sinvalocho M.A. with ethyl methanesulfonate, in which the disease- resistance response is simulated in the absence of any pathogen, and a spontaneous HR-like response associated with cell death and enhanced resistance to leaf rust are observed [37]. The M66 mutant originated from X-ray mutagenesis of the Guardian wheat cultivar, and shows increased resistance to powdery mildew and yellow rust [38]. LMMs also display significant adult-plant resistance to leaf rust compared with non-LMMs, and *lm* has been localized between either the marker *Xwmc85*.*1* or *Xgwm264*.*1* and the centromere of chromosome 1B [12]; The LM phenotype characterized in recombinant inbred lines of Yanzhan1/Zaosui30, results from the interaction of two recessive genes derived from each parent, which have been mapped to chromosomes 3BS and 4BL. The observed lesions are light-dependent and have negative effects on subsequently developing traits and the enhanced expression of resistance to leaf rust [13]. In addition, the lesion symptoms of both AIM9 [39] and LF2010 [14] are controlled by a single recessive gene, and the total chlorophyll content and net photosynthetic rates of these plants are reduced with the onset of symptoms in the leaves. LF2010 is a light- and temperature- affected LMM, whose plant height, spike length, spike number per plant, grain weight per plant, grain number per spike, seed setting rate, and flag leaf length are lower compared with wildtype plants [14].

An F_1_ individual derived from a 3-1/Jing411 cross in 2000 was identified as an LMM, and after being backcrossed six times using 3–1 as the recurrent parent and self-pollinated twice, this mutant was designated lm3. In the present study, we described the *lm3* mutant exhibiting spontaneous cell death, and provided evidence that PCD and ROS accompanied the formation of lesion spots. The mutant phenotype is light dependent, as revealed through different light intensities and photoperiods, and a panel of defense-related genes was up-regulated during the initiation of lesion formation, resulting in the resistance to the powdery mildew fungus. We mapped the mutant *lm3* gene to a narrow region on the long arm of chromosome 3B using the Chinese Spring genome sequence.

## Materials and Methods

### Plant material

The LMM was obtained at the Experimental Station (ES) of the Institute of Genetics and Developmental Biology, Chinese Academy of Sciences (IDGB, CAS), in May 2000. During anthesis, one plant among the 30 F_1_ plants derived from the 3-1/Jing411 cross exhibited LM leaves. Jing411 is a commercial winter wheat variety released in 1991 in Beijing, China, whereas 3–1 is an advanced breeding line bred in the 1990s at China Agricultural University, Beijing. The mutated F_1_ was backcrossed six times with the female parent 3–1 in the subsequent three years, and the necrotic symptoms in the progenies of each backcrossing generation were selected. The BC_6_ progeny was subsequently self-pollinated twice, and one plant with the necrotic symptom phenotype was selected and designated as lm3 in 2005, which resembled the initial maternal parent 3–1 plant, except for the necrosis phenotype. Thus, lm3 and 3–1 could serve as a set of near-isogenic lines for the investigation of related gene expression in the LM. Both lines were grown at the ES of IDGB, CAS during the 2005–2006 cropping season for further analysis.

### Measurement of chlorophyll contents

Chlorophyll *a* and *b* contents were measured using the flag leaves of lm3, 3–1 and the associated F_1_ plants two weeks after the anthesis stage [40]. The flag leaves of lm3 at this stage were fully scattered with lesions. Flag leaf samples of ~200 mg each were cut into pieces, and subsequently soaked in 20 mL of 80% acetone at 4°C for 48 h in darkness until the leaf samples became colorless. A solution of 80% acetone was used as a blank. The absorbance values at wavelengths of 663- and 645- nm were measured using the chlorophyll extract and a spectrophotometer (Shimadzu UV-1700). Three biological repeats were assayed for each sample.

### Light-dependent analysis

lm3 and 3–1 seeds were surface-sterilized in 10% hydrogen peroxide (H_2_O_2_) for 10 min, rinsed with Millipore water, and germinated on moist filter paper for 2 d at 25°C. Uniformly sized seedlings were transplanted to 1000 mL rectangular plastic pots containing 600 mL of half-strength aerated Hoagland nutrient solution. The nutrient solution was refreshed every three days. Wheat seedlings were grown in a greenhouse under a day/night temperature regime of 22–25°C and normal 50% air moisture. Illumination was provided by cool-white fluorescent lamps with photosynthetically active radiation of 200 μmol m^−2^ s^−1^ (*μE*) under a 16 h photoperiod. The treatments involving different light intensities and day lengths were performed on the 8^th^ day, when the plants exhibited a fully developed first leaf and expanding second leaf. The distance of the seedlings from the lamps was adjusted to provide a 50% light intensity (100 *μE*), and 6 h and 12 h day lengths were controlled by turning off the lamps. Sections of the second newly expanded leaves were covered with aluminum foil to completely reflect incident light. After lesions on the 3^rd^ leaves of lm3 plants had formed completely under the conditions of 200 *μE* with a 16 h photoperiod, the lesions on all leaves were carefully checked, including the regions covered with aluminum foil and the leaves of 3–1 plants.

### Histochemical analysis

Fresh leaf samples were collected from LM plants and wildtype plants during the heading stage, and trypan blue staining [41] was performed to detect cell death in LM plants. The leaves were immersed in a 70% lactic acid-phenol- trypan blue solution [2.5 mg/ml trypan blue, 25% (w/v) lactic acid, 23% water-saturated phenol, 25% glycerol, and H_2_O], and the leaf samples were then heated in boiling water for 15 min, and stained for 12 h at room temperature. The leaf samples were finally stored in chloral hydrate solution (25 mg in 10 mL H_2_O) for destaining and subsequently photographed.

Hydrogen peroxide (H_2_O_2_) was detected in leaves using 3,3’- diaminobenzidine (DAB) staining [42]. Leaf samples were submerged in 1mg/mL DAB (pH = 3.8), and incubated in a growth chamber for 8 h to allow DAB uptake and reaction with H_2_O_2_; then, they were transferred to 95% ethanol and heated in boiling water for chlorophyll extraction. Finally, the leaf samples were stored in fresh ethanol at 25°C until chlorophyll had disappeared, and examined using scanner (Image Scanner III).

### Lesion-related gene expression analysis

Relative quantitative reverse transcription polymerase chain reaction (RT-qPCR) was performed with gene-specific primers (Table 1) [43], designed based on wheat sequences, to assess the transcript abundances of lesion-related genes during the development of necrotic spots. Tissue samples obtained from lm3 and 3–1 seedlings and cultured with half strength Hoagland nutrient solution in the greenhouse (200 μmol m^−2^ s^−1^ of photosynthetically active radiation, 16 -h photoperiod, 22–25°C day/night temperature regime, and 50% air moisture), were used to assay the expression levels of pathogen-related genes. When symptoms of lesions were visible on the 3^rd^ leaf tips of lm3 plants, samples of the 1^st^ leaf (fully developed lesions), 3^rd^ leaf (developing lesions) and the roots (no lesions) were individually collected and immediately frozen in liquid nitrogen, and were then stored at −80°C for further RNA extraction.

**Table 1:** Details of the putative defense genes and primer sequences used for RT-PCR.

Accession	Gene name	Protein family	Forward primer (5'-3')	Reverse primer (5'-3')
AJ007348	*PR1*.*1*	PR1 (basic)	CTGGAGCACGAAGCTGCAG	CGAGTGCTGGAGCTTGCAGT
Y18212	*PR2*	PR2	CTCGACATCGGTAACGACCAG	GCGGCGATGTACTTGATGTTC
AB029934	*Chitinase 1*	PR3	AGAGATAAGCAAGGCCACGTC	GGTTGCTCACCAGGTCCTTC
AJ006098	*Wheatwin 1*–*2*	PR4	CGAGGATCGTGGACCAGTG	GTCGACGAACTGGTAGTTGACG
X56011	*TaPERO* (*Peroxidase*)	PR9	GAGATTCCACAGATGCAAACGAG	GGAGGCCCTTGTTTCTGAATG
CA684431	*PR10 homolog*	PR10	TTAAACCAGCACGAGAAACATCAG	ATCCTCCCTCGATTATTCTCACG
CD863039	*PWIR2*	Thaumatin-like	AGGTAATTTTTTTATTGCCCTGTACTG	TTACAGCCGCCGTACTACATGT
AJ237942	*TaGLP2a*	Germin-like	AACAAAGGTGATGTGTTCGTCTTC	GAGCCGGTCTATTGTATTCTTTTCC
U32428	*WCI2*	lipoxygenase	TAGGAACTGGAACTTCACCGAGC	GGTAGTCCTTGATGTGCAGCGAC
U32429	*WCI3*	sulfur-rich/thionin-like protein	AAAGTTGGTCTTGCCACTGACTG	TCGACAAAGCACTTCTGGATTTC
Ta2776	RNase L inhibitor-like	68 kDa protein HP68	CGATTCAGAGCAGCGTATTGTTG	AGTTGGTCGGGTCTCTTCTAAATG

Total RNA was isolated using TRIZOL reagent (Sigma Chemical Co., USA) according to the manufacturer’s instructions, and DNase treatment (Invitrogen, USA) was performed on the crude RNA preparations, followed by phenol/chloroform extraction [44]. Complementary DNA was synthesized using a combination of 5 ng/μL oligo(dT) and 2.5 ng/μL random hexamers for priming, employing the SuperScript II First-Strand Synthesis System (Invitrogen, USA) according to the manufacturer’s instructions. Each 20 μL sample of synthesized cDNA was diluted 5-fold with nuclease-free water and 2 μL of this diluted cDNA was used for each amplification reaction in the RT-qPCR analysis. RT-qPCR was performed in optical 96-well plates using the Roche LightCycler 480 Detection System (Roche, USA). Each reaction contained 5 μL of 2X SYBR Green Ⅰ Master Mix (Roche, USA), 250 nM each forward and reverse gene-specific primer and 2 μL of the cDNA template. The PCR program consisted of a pre-denaturation step at 95°C for 5 min, followed by 45 cycles at 95°C for 10 s, 60°C for 10 s and 72°C for 10 s, with a final step at 95°C for 1 min and 40°C for 5 min. Three biological replicates of each sample together with two technical replicates were examined and the data were retained only when the standard deviation between replicates was ≤0.3 Ct. Ta2776 (RNase L inhibitor-like protein) [45], amplified using the forward primer 5’-CGATTCAGAGCAGCGTATTGTTG-3’ and the reverse primer 5’-AGTTGGTCGGGTCTCTTCTAAATG-3’, was selected as the reference gene after a survey of 16 genes among various samples. The corresponding fold-change in the expression of the respective genes was calculated according to the threshold cycle (Ct) using the △△Ct method with LightCycler Data Analysis Software (Roche, USA).

### Evaluation of powdery mildew resistance

The LM and wildtype plants were evaluated for their response to powdery mildew under natural infection in the field at the ES of IDGB, CAS during the 2009–2015 cropping seasons, and in the greenhouse in the winter of 2015. LM and wildtype plants were grown randomly with two replications. At the anthesis stage, a mild-to-severe powdery mildew epidemic usually occurred in the field at the ES of IDGB, CAS.

The powdery mildew response of lm3 was also assessed following artificial inoculation in a growth chamber. The single spore-derived isolate, *Bgt* E18 was inoculated by shaking conidiospores from susceptible Kenong199 plants onto adult lm3 and wildtype plants. For each line, approximately five seeds were planted in a 25 cm pot as one replicate, and three replicates were grown in the greenhouse until the anthesis stage, at which time the pots were transferred to the growth chamber for inoculation. Reactions were scored 10 days after inoculation, when the wildtype plants were clearly infected.

### Genetic analysis

Two commercial winter wheat varieties, Jingdong8 and Nongda3291, which were released in Beijing in 1995 and 2001, respectively, were crossed as the male parents with lm3 in 2005. The F_1_ progenies of the lm3/Nongda3291 cross were self-pollinated or backcrossed with Nongda3291 in 2006, and the necrotic symptoms in the F_2_ and BC_1_ populations were measured during the 2006–2007 cropping season at the ES of IDGB, CAS. A second F_2_ population derived from the lm3/Jingdong8 cross was also screened during the same season. The necrotic symptoms were scored at the anthesis stage and recorded as the presence (1) or absence (0) of the lesions on the flag leaf. The F_2_ population derived from the lm3/Jingdong8 cross was employed for mapping the LM phenotype, and the derived F_7:8_ segregating population was for fine-mapping to the 3B chromosome of Chinese Spring.

### Genomic DNA extraction and marker analysis

The F_2_ population derived from the lm3/Jingdong8 cross was subjected to mapping the LM on a wheat chromosome. The young flag leaves of each F_2_ were collected in liquid nitrogen and ground to a fine powder using a GenoGrinder 2000 homogenizer (Spex CertiPrep, USA) for DNA isolation using the CTAB method [46]. Bulked segregant analysis [47] was performed by two bulk DNA pools containing equal amounts of DNA from 10 F_2_ plants with or without the lesion symptoms. A total of 729 SSR primers, including BARC [48], CFA and CFD [49, 50] covering all 21 wheat chromosomes, were screened between the two parents (lm3 and Jingdong8), and the polymorphic primers between the parents were used to screen the bulks. The polymorphic primers between the bulks were subsequently exploited to genotype the entire F_2_ population, and the available SSR markers located on chromosome 3B (http://wheat.pw.usda.gov/GG2/index.shtml) were used to screen the F_2_ population after the markers on 3B were associated with the lesion symptoms. The PCR reaction for SSR was conducted using a Veriti Thermal Cycler (Applied Biosystems, Foster City, CA, USA) in a total volume of 20 μL containing 100 ng of template DNA, 0.6 U of Taq polymerase (Takara Bio, Otsu, Japan), 1× PCR buffer, 2.0 mM MgCl_2_, 0.2 mM dNTP and 0.2 μM forward and reverse primers. The amplification program included pre-denaturation for 5 min at 95°C, followed by 35 cycles at 94°C for 30 s, 50–60°C (depending on SSR primers) for 45 s and 72°C for 45 s, and a final extension step at 72°C for 10 min. The PCR products were mixed with 5 μL of loading buffer (98% formamide, 0.3% of each bromophenol blue and xylene cyanol and 10 mM EDTA), denatured at 95°C for 5 min and chilled on ice until further use. Each sample (5 μL) was loaded on a pre-heated 4.5% denatured polyacrylamide gel. Electrophoresis was performed at 75 W for 45 min using a BIO-RAD PowerPac 3000 in 1× TBE buffer. Gels were subsequently silver-stained according to the protocol of Bassam [51].

**Fig 1:**
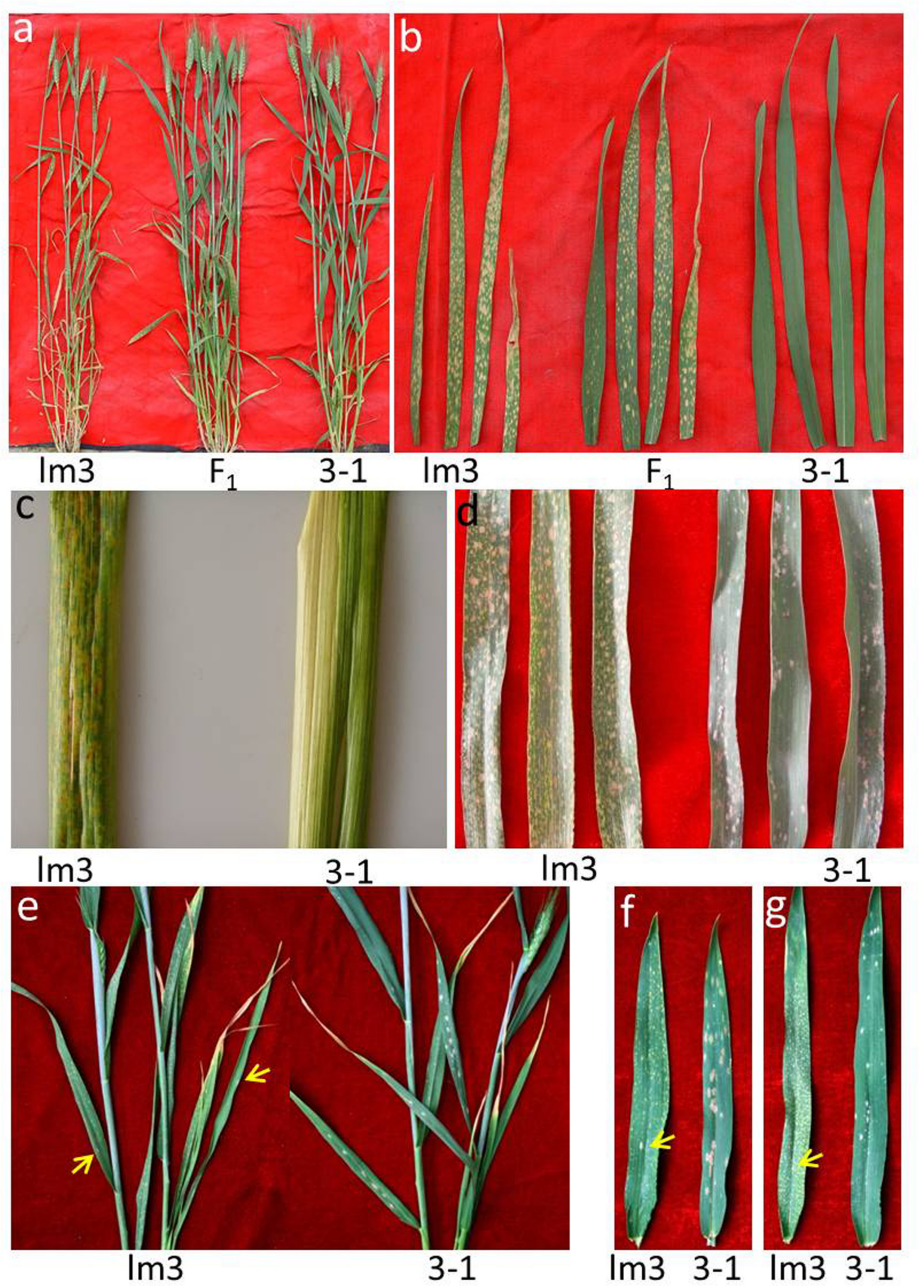
Phenotype of the lm3 mutant. **a** Phenotype of the lm3 mutant, the wildtype (3–1) plants and their F_1_ progeny at two weeks after anthesis in the field. **b** Comparison of lesion symptoms in different leaves in the lm3 mutant, the wildtype (3–1) plants and their F_1_ progeny. For each line, the top fourth, top third, top second and flag leaves are presented from left to right. **c** Lesion symptoms on the leaf sheath of the lm3 mutant, and wildtype (3–1). **d-g** Enhanced resistance to powdery mildew. **d** Reaction of the lm3 mutant and wildtype (3–1) plant to *Blumeria graminis* f. sp. *tritici* (*Bgt*) under natural infection in the adult plant stage under field conditions in 2009. The flag leaves of lm3 plants were covered with yellow necrotic spots, but no spores of *Bgt* were visible, while many white and brown powdery mildew spores were present on the wildtype (3–1) plants. **e** Powdery mildew reaction under natural infection at the adult plant stage in the greenhouse in 2015. Only two instances of *Bgt* sporulation were observed on the older leaves of lm3 plants (yellow arrows), but many white powdery mildew spores were visible on the wildtype (3–1) plants. **f** and **g** Responses of the lm3 mutant and wildtype (3–1) plants to *Bgt* E18 in the growth chamber at the adult stage in 2015. Many white and brown spores of *Bgt* were observed on the flag leaves (**f**, *right*) and top second leaves (**g**, *right*) leaves of wildtype (3–1) plants, whereas only one spore (yellow arrows) was present on each of the corresponding leaves of the lm3 mutant (**f** and **g**, *left*), where necrotic spots were widely distributed. All the visible brown spots on lm3 leaves and sheath are necrotic lesions, except a few spores of *Bgt* indicated by yellow arrows on lm3 leaves (**e**, **f** and **g**), while most visible whitish spots on the leaves of wildtype (3–1) plants are spores of *Bgt* on the panels (**d**, **e**, **f** and **g**).

For fine mapping, twenty F_7_ individuals derived from the lm3/Jingdong8 population were self-pollinated and selected based on mild necrotic symptoms on the flag leaves at the anthesis stage (Fig 1B), in which the mild symptoms indicate the heterozygosity of the gene conferring necrotic symptoms. These F_7:8_ lines were grown during the 2014–2015 cropping season at the ES of IDGB, CAS. The DNA from each individual was isolated from young seedling leaves, and necrotic symptoms were screened on flag leaves at the anthesis stage. Because *lm3* is located on chromosome 3B, flanked by *Xbarc1140* and *Xbarc268*, and co-segregated with *Xbarc203*, these SSR markers were anchored to the 3B genome of Chinese Spring (https://urgi.versailles.inra.fr/gb2/gbrowse/wheat_annot_3B/) at 374.423 Mb (BV211508, *Xbarc1140*), 511.906 Mb (BV211642, *Xbarc203*), and 549.106 Mb (BV211841, *Xbarc268*). Additional SSR markers from regions 400–550 Mb on chromosome 3B were developed according to the sequences from Chinese Spring (https://urgi.versailles.inra.fr/gb2/gbrowse/wheat_annot_3B/). SSRLocator [52], a microsatellite search tool, was subsequently applied to identify SSR sequences using default parameters, and the flanking regions were used to design primers with Primer3 in batch mode with the assistance of the SSRLocator interface module. All SSR primers were commercially synthesized at Beijing Biomed Genetechnology Company Ltd. for further PCR analysis.

### Data analysis

The data on the scores of the lesion symptoms in the three populations and the SSR marker genotypes were subjected to a Chi-square test to determine the goodness of fit to the expected ratio using SPSS software version 17 (SPSS Inc., Chicago, IL, USA). A genetic linkage map was constructed using MAPMAKER/EXP ver. 3.0 [53], employing a logarithm odds value of 3.0 as the threshold. Recombination frequencies were converted to centi-Morgans (cM) using the Kosambi function [54], and a genetic map was drawn using Mapchart v2.2 software [55]. All statistical analyses applied in the transcript abundance analysis of lesion-related genes were conducted using Microsoft Excel (Microsoft Crop., Redmond, WA).

## Results

### Occurrence of lesion symptoms in lm3

**Fig 2:**
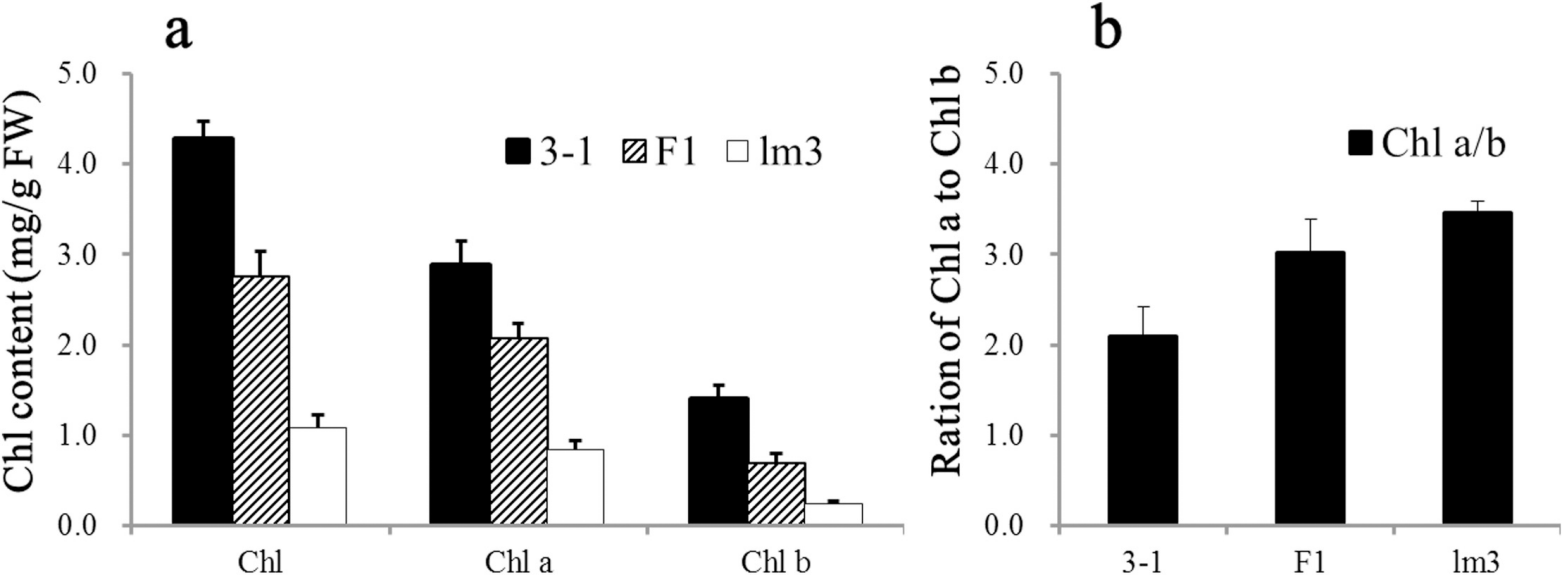
Chlorophyll content and ratio in the leaves of 3–1, lm3 and their F_1_ progeny. **a** Chl a, Chl b and total Chl content in 3–1, lm3 and their F_1_ plants. **b** Ratio of Chl a to Chl b in 3–1, lm3 and their F_1_ plants. The means and SD (standard deviation) are shown with statistical analysis. Chl, chlorophyll; Chl a, chlorophyll *a*; Chl b, chlorophyll *b*; FW, fresh weight.

Lesion symptoms were observed in an F_1_ plant derived from 3-1/Jing 411 at the anthesis stage in the field during 2000. The mutated F_1_ was backcrossed six times with the female parent 3–1 and self-pollinated twice. One progeny was selected and designated as lm3 in 2005, resembling 3–1 except for the lesion symptoms. The lesion symptoms in lm3 were thoroughly examined for more than three growing seasons. The young seedlings exhibited small (1–3 mm), discrete white lesions in the absence of pathogens, and the lesions became more obvious at booting stage and merged into large necrotic spots at later stages (Fig 1B). The lesions progressively developed from the lower to upper leaves, and the lower leaves showed more severe symptoms of necrosis (Fig 1A). On a specific leaf, the lesions were observed at the tip and expanded to the middle and bottom of the leaf blade to the leaf sheath. The lesions were not observed in other tissues except the leaf blade and leaf sheath (Fig 1C). The older leaves with lesions turned yellow and showed early senescence. Eventually, the entire plant dried up earlier than normal plants (Fig 1A). With the lesion formation, the total chlorophyll content in the *lm3* mutant significantly decreased, showing a 74.90% reduction of which the contents of both Chlorophyll *a* and *b* were distinctly decreased (Fig 2A). In the *lm3* mutant, the Chlorophyll *a* content was 0.83 mg/g, showing a 71.07% reduction compared with wildtype, whereas the Chlorophyll *b* content decreased by 82.77%, resulting in a higher Chlorophyll *a*/*b* ratio (3.46 vs. 2.09) in the *lm3* mutant. Moreover, the reduction in the Chlorophyll *a* content was slower than that of the Chlorophyll *b* content in F_1_ plants between lm3 and 3–1, resulting in a Chlorophyll *a*/*b* ratio of 3.02, intermediate between that of 3–1 and lm3 (Fig 2B). These findings indicated that Chlorophyll *b* was more prone to damage than Chlorophyll *a* during lesion formation.

### Light activates the lesion mimic phenotype

**Fig 3:**
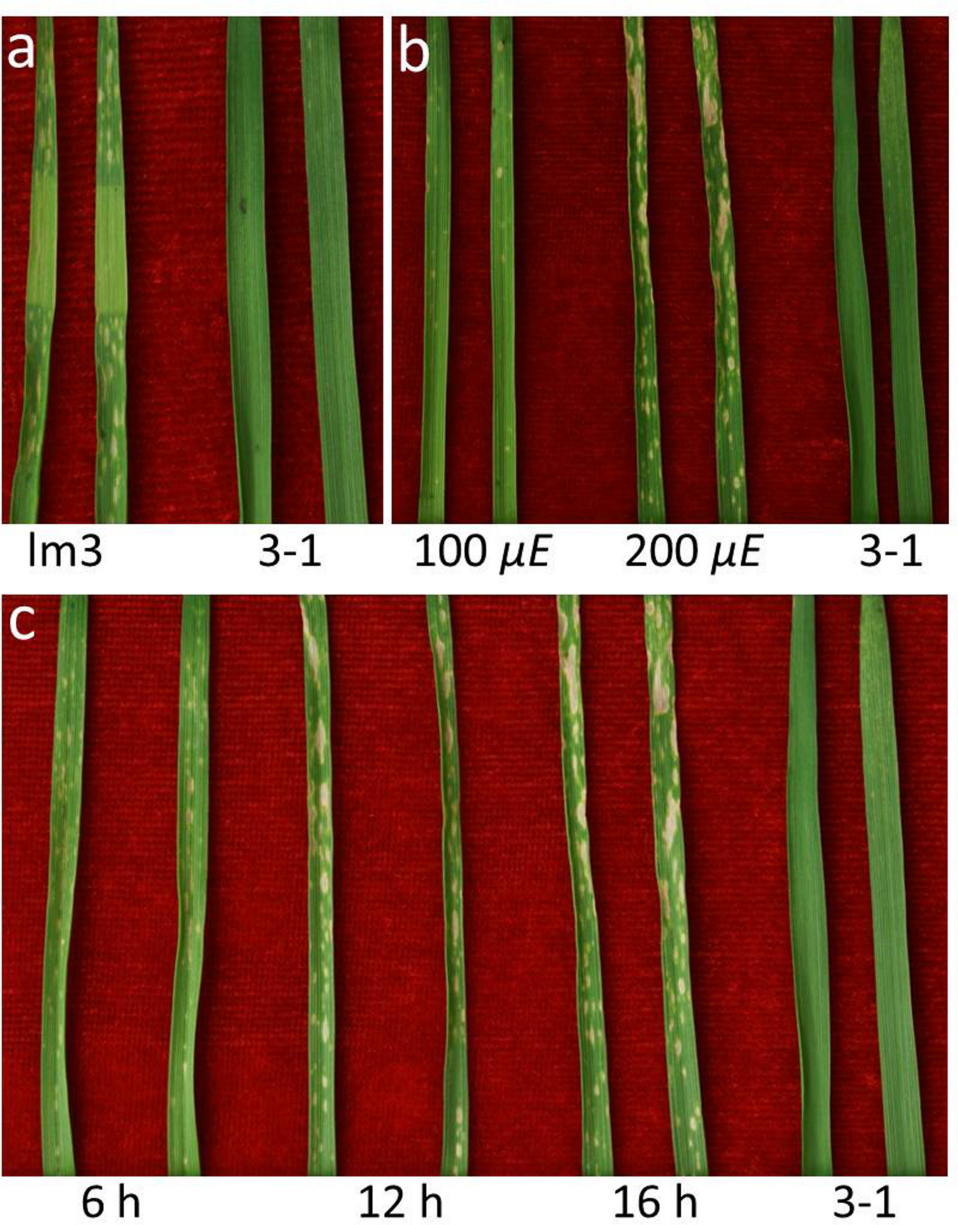
Light dependence of lesion initiation. **a** No lesion spots were observed in the middle segment of the leaves in the *lm3* mutant, which was hidden from the light. **b** Effect of light intensity on lesion formation. Fewer lesion spots were observed under the treatment with 100 μmol m^−2^ s^−1^ (μ*E*) compared with 200 μ*E* in the *lm3* mutant. **c** Effect of photoperiod on lesion formation. Several larger lesion spots were observed on the lm3 leaves after a longer day-length treatment. Two representative leaves are shown for each treatment, and the samples on the right are shown as the wildtype plant (3–1) in each panel.

Light has been reported as an important stimulus in some LMs [13, 56–59]. Therefore a shading assay was performed in the greenhouse of IDGB, CAS to test whether lesion formation in the *lm3* mutant was dependent on light. Leaves were covered with aluminum foil to block light before the expression of lesions, and 2 weeks later, no lesions were observed on the covered portions of the lm3 leaves, whereas necrotic spots were scattered throughout the bare areas under the normal illumination treatment (Fig 3A), which revealed that light plays an important role in lesion formation.

We speculated that the *lm3* mutant might be sensitive to the light intensity because seedlings at the same stage grown in late autumn and early spring showed a different severity of lesion symptoms. To investigate the involvement of light in lesion formation, various photoperiod and light intensity treatments were applied to lm3 seedlings cultured with half-strength Hoagland solution in the greenhouse. Under the condition of 200 μ*E* and a 16 h photoperiod, lesions first appeared as small necrotic spots on the leaves and later expanded to form larger lesions with increasing incubation time (Fig 3C). Compared with the lesions observed under a 16 h photoperiod, the number of large lesions (>3 mm) was visibly decreased under a 12 h photoperiod, an even more so under a 6 h photoperiod, though the number of lesions did not change significantly (Fig 3C). The light intensity also influenced lesion formation (Fig 3B). Under the conditions of a 50% light intensity (100 μ*E*) and a 16 h photoperiod, the size and number of lesions decreased significantly; large lesions (>3 mm) were not observed and most of the visible lesions were very small (<1 mm) with the total number of lesions being reduced to less than 20% of that under a 100% light intensity with a 16 h photoperiod. These results indicated that lesion symptoms were more easily stimulated by the light intensity treatments than the photoperiod treatments. The severity of lesion formation was also noted with the emergence of leaves in the *lm3* mutant, and the older leaves were found to exhibit a greater severity of lesion formation (Fig 1B). These findings suggested that the lm3 lesion were induced by light, but both the light intensity and day length modulated lesion formation (Fig 3).

### Programmed cell death and H_2_O_2_ accumulation

**Fig 4:**
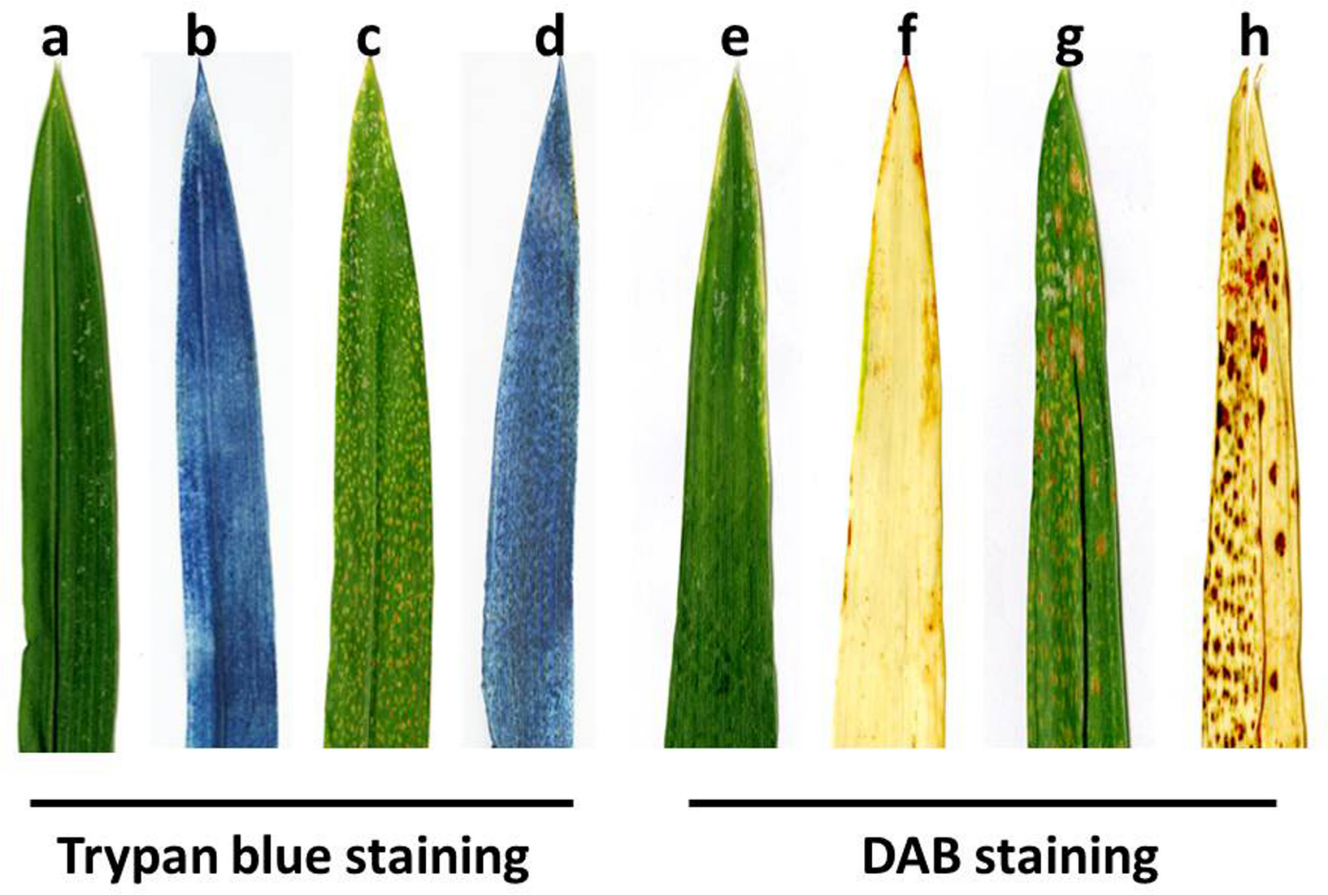
Histochemical analysis of wildtype and lesion-mimic plants. **a-d** Trypan blue staining for cell death; **e-h** DAB staining for H_2_O_2_ accumulation. **a, e** Phenotype of the wildtype before Trypan blue and DAB staining; **b, f** Phenotype of the wildtype after Trypan blue and DAB staining; **c, g** Phenotype of the lesion mutant prior to Trypan blue and DAB staining, in which the brown spots are the lesions in the *lm3* mutant; **d, h** Phenotype of the lesion mutant after Trypan blue and DAB staining, in which the dark blue (**d**) and reddish-brown spots (**h**) show the cell death and ROS enriched areas in the *lm3* mutant, respectively. The data represent at least three experiments.

Programmed cell death (PCD) and reactive oxygen species (ROS) are usually accompanied by necrotic spot formation [1]. Trypan blue staining assay is a traditional method for staining dead tissues or cells and irreversible membrane damage [60]. After Trypan blue staining, the leaves of the *lm3* mutant exhibited a deep blue color at the site of lesions, whereas the surrounding normal cells in the *lm3* mutant and wildtype leaves exhibited negative Trypan blue staining (Fig 4A–4D), suggesting that PCD occurred during lesion formation in the *lm3* mutant. PCD was also demonstrated by a DNA laddering during lesion formation (data not shown). To confirm that ROS accompanied PCD, we performed a DAB staining assay to assess H_2_O_2_ accumulation, in which a reddish-brown polymer precipitate is generated by the interaction between DAB and H_2_O_2_ in the presence of peroxidase [61]. After staining, a large amount of reddish-brown precipitate was only observed at necrotic sites in the *lm3* mutant leaves (Fig 4E–4H), and dark reddish-brown staining appeared with increasing severity of necrosis (Fig 4F and 4H), indicating a high level of H_2_O_2_ accumulation in the *lm3* mutant. This finding indicated that ROS accumulation in cells might be responsible for cell death and lesion formation, and the histochemical staining assay confirmed that the *lm3* mutant suffered from a hypersensitive reaction and exhibited programmed cell death with a visible phenotype at necrotic sites.

### Wheat defense genes induced through LM

**Fig 5:**
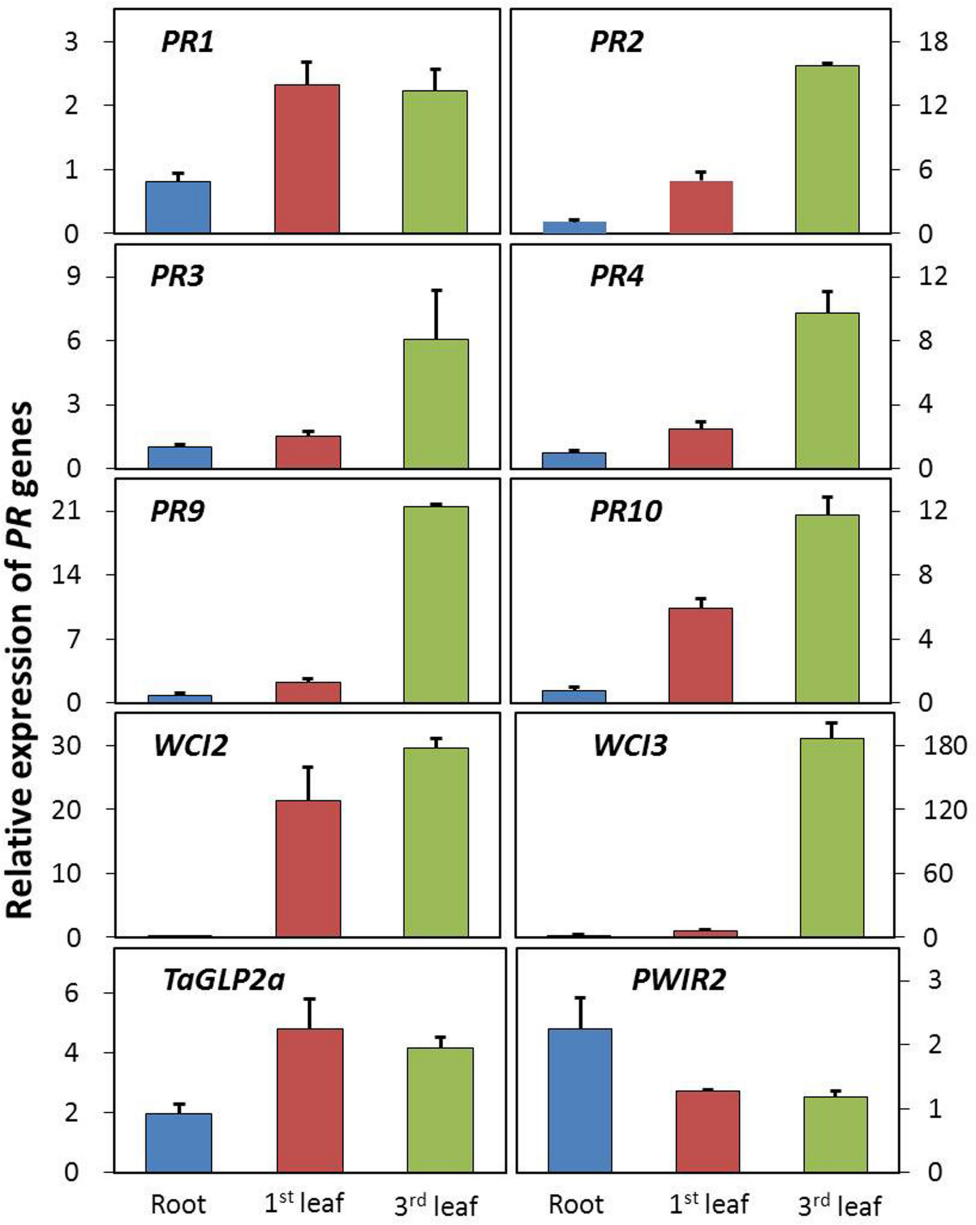
Transcript abundance of defense-related genes during lesion formation. Transcript abundance was determined via RT-qPCR, in samples of the roots (no lesions), the 1^st^ leaves (fully developed lesions), and the 3^rd^ leaves (developing lesions) of lm3 plants and corresponding tissues of 3–1 plants, and the fold-changes, corresponding to the relative expression of defense-related genes are shown on the *y*-axis, based on normalization of the expression data for lm3 to 3–1. The *error bars* represent the standard deviation between biological replicates. Mean fold-changes in the transcript abundance calculated using the △△*C*t method between biological replicates ± standard deviation.

LMMs are widely considered to be involved in defense responses in plants. The expression of defense genes is strongly correlated with the onset of lesion formation in both dicots and monocots [62]. To evaluate whether lesion formation is correlated with the expression of defense-related genes, total RNA was isolated from samples of the 1^st^ leaves (fully developed lesions), 3^rd^ leaf (developing lesions) and roots (no lesions) of the *lm3* mutant, and corresponding tissues in WT (3–1). A set of wheat defense-related genes was selected, including *wheat chemically induced* (*WCI*) and *PR* genes based on the involvement of these genes in defense responses to fungal pathogens in wheat [63], and primers were designed based on wheat sequences to amplify all three orthologous copies of each defense-related gene in the hexaploid wheat genome (Table 1). Using RT-qPCR, changes in the transcript levels of these genes in the *lm3* mutant relative to those in WT plants were determined in root, 1^st^ leaf, and 3^rd^ leaf tissues, with the reference gene, Ta2776 (RNase L inhibitor-like protein), which was evaluated using various samples from a panel of candidate reference genes [45]. The RT-qPCR data revealed similar patterns of transcript abundance for seven of the ten selected defense-related genes in three assayed tissues in the *lm3* mutant compared with WT. No significant changes in root samples but slight up-regulation (1.5–6.1-fold, except for the 21.4-fold up-regulation in *WCI2*) in the 1^st^ leaf, and dramatic up-regulation (6.1–186.1-fold) in the 3^rd^ leaf of the *lm3* mutant were observed in defense-related genes, *PR2*, *PR3*, *PR4*, PR9, *PR10*, *WCI2* and *WCI3* (Fig 5). When the fold-change between the 1^st^ and 3^rd^ leaves was compared in the *lm3* mutant and WT plants, all seven genes were found to exhibit a stronger response to lesion formation, particularly *PR9* and *WCI3*, which presented 9- and 30-fold increases, respectively. This finding revealed that these defense-related genes were induced during lesion formation, and their up-regulation significantly decreased once the lesions had fully developed. Contrast to the seven genes indicated above, the up-regulation of *PR1* and *TaGLP2a* differed only slightly between the 1^st^ and 3^rd^ leaves of the *lm3* mutant (Fig 5). The transcript abundance of *PR1* in the 1^st^ and 3^rd^ leaves was approximately two-fold higher, while that of *TaGLP2a* was four-fold higher in the *lm3* mutant compared with WT, suggesting potential preservation of the constitutive activation of a defense signaling pathway after lesion initiation in the *lm3* mutant. In contrast, the transcript abundance of *PWIR2*, a thaumatin-like protein induced in plant defenses during pathogen attack [63], remained unaltered during lesion formation. However, the expression of this gene was unexpectedly found to be induced two-fold in the root samples from the *lm3* mutant, although no lesions were detected in the roots. This phenomenon was also observed for *WCI3* and *TaGLP2a* (Fig 5), suggesting that root tissues might respond to lesion formation in the shoots.

### Enhanced resistance to powdery mildew pathogens

Many LMMs of *Arabidopsis* and rice show various levels of defense resistance [10, 17, 20, 28, 62]. To determine whether the *lm3* mutant is resistant to powdery mildew, natural powdery mildew infections were evaluated at the anthesis stage in field-grown 3–1 and lm3 plants during the 2009–2015 cropping seasons. In most seasons, sporulation was widely observed on the leaves of wildtype plants, indicating an epidemic of powdery mildew and showing the high susceptible of 3–1 plants to powdery mildew pathogens, whereas spores were not observed on the flag leaves of lm3 plants undergoing lesion formation (Fig 1D). This phenomenon was also observed in adult lm3 and wildtype plants in the greenhouse (Fig 1E), which suggested resistance to powdery mildew during lesion formation in the *lm3* mutant. However, a small amount of sporulations appeared on older leaves with lesion spots in *lm3* mutant plants in later stages (Fig 1E). To confirm the observed resistance, both lm3 and 3–1 adult plants were inoculated with the wheat powdery mildew fungus *Blumeria graminis* f. sp. *tritici* (*Bgt*) in a growth chamber. At 10 days after inoculation with *Bgt* E18, the wildtype, 3–1 plants were heavily infected with large areas of white and brown spores on the flag leaves (Fig 1F) and top second leaves (Fig 1G), whereas few spores were observed in the *lm3* mutant, despite the many lesions on its leaves. These data demonstrated the significantly enhancement of powdery mildew resistance in the *lm3* mutant during lesion formation (Fig 1).

### Genetic control of the lesion mimic phenotype

To investigate the inheritance of the LMM, two crosses (viz. lm3/Nongda3291, and lm3/Jingdong8), were performed, and the resultant F_1_ plants showed lesion symptom on the leaves, but the speed and severity of lesion formation were reduced compared with homozygous *lm3* plants, suggesting that the *lm3* LM phenotype was genetically partial dominant. The genetic segregation was investigated using two F_2_ populations derived from the lm3/Nongda3291 and lm3/Jingdong8 crosses. In these two F_2_ populations, 376 and 181 plants showed *lm3* phenotypes, and 119 and 66 plants exhibited WT phenotypes, respectively. The segregation ratios in both F_2_ populations were consistent with the expected segregation at a single dominant locus (Table 2). This genetic mode was confirmed in the BC1 population derived from the lm3/Nongda3291 cross and backcrossed with Nongda3291, in which 265 and 282 plants had lesions and no lesions on their leaves, respectively (Table 2).

**Table 2:** Segregation analysis of the LM symptoms in three segregating populations in 2007.

Cross	Population	LM phenotype^a^	Normal phenotype^a^	Expected ratio^b^	χ^2^^c^	P-value^d^
lm3/Nongda3291	F2	376	119	3:1	0.243	0.622
lm3/Nongda3291	BC1	265	282	1:1	0.528	0.467
lm3/Jingdong8	F2	181	66	3:1	0.390	0.532

a Number of individuals in populations with or without lesion symptom.

b Expected Mendelian single gene segregation ratio (LM:Normal)

c Calculated Chi-square (**χ**^2^)

d Likelihood that the observed segregation ratio does not fit a 3:1 or 1:1 ratio.

### Molecular mapping of the *lm3* locus

**Fig 6:**
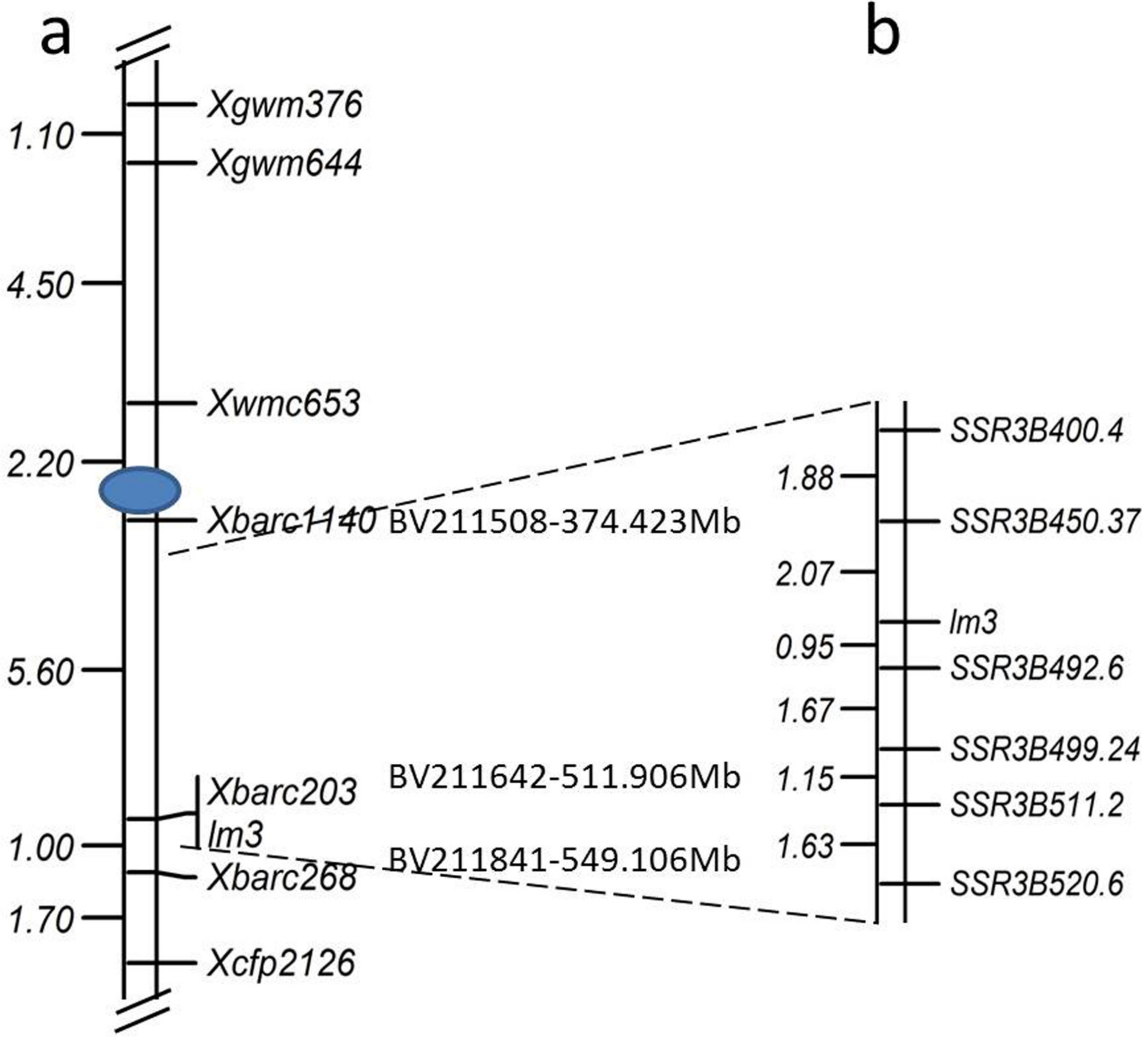
Genetic map of the region surrounding *lm3* on chromosome 3B. **a** Map constructed using the DNAs from 190 individual F_2_ plants from a cross between lm3 and Jingdong8. **b** Detailed map of *lm3* on chromosome 3B developed using the derived segregating population from the lm3/Jingdong8 cross, and the genome sequence of Chinese Spring. a and b are not in the same portrait.

The *lm3* locus was genetically mapped using the genomic DNA from 190 individual F_2_ plants derived from the lm3/Jingdong8 cross and their corresponding LM phenotype. A total of 729 SSR primers distributed over all 21 chromosomes in hexaploid wheat were screened, and ~150 SSR markers exhibited polymorphisms between lm3 and Jingdong8. The bulked segregant analysis of these polymorphic markers was applied to assay the mutant and WT pools, in which equal amounts of DNA from 10 F_2_ individuals with or without lesion symptoms were pooled. Only two markers, *Xbarc203* and *Xbarc268*, both located on chromosome 3B, were detected as polymorphic between the two parents and the lesion and WT bulks. These two polymorphic markers were closely linked with the lesion symptom after screening all 190 F_2_ individuals derived from the lm3/Jingdong8 cross. To confirm these data, we performed the linkage analysis of *Xbarc203* and *Xbarc268*, along with five other SSR markers located on chromosome 3B exhibiting polymorphisms between lm3 and Jingdong8, and eventually a linkage map including seven polymorphic markers and the *lm3* locus was constructed (Fig 6A). The *lm3* locus co-segregated with *Xbarc203* and was flanked by *Xbarc1140* and *Xbarc268*, where *Xbarc1140* is 5.6 cM proximal to the *lm3* locus, and *Xbarc268* is 1.0 cM distal to the locus.

To fine map the *lm3* locus, the derived F_7:8_ segregating population from a lm3/Jingdong8 cross with 5,376 individuals was subjected to DNA extraction and phenotyping at the ES of IDGB, CAS, and the 3B genome sequences of Chinese Spring (https://urgi.versailles.inra.fr/gb2/gbrowse/wheat_annot_3B/) were exploited for SSR marker development. Because *Xbarc1140*, *Xbarc203*, and *Xbarc268* were located at positions 374.423 Mb (BV211508, *Xbarc1140*), 511.906 Mb (BV211642, *Xbarc203*), and 549.106 Mb (BV211841, *Xbarc268*), respectively, additional SSR markers were developed from the 400–402 Mb, 450–452 Mb, 479–481 Mb, 492–494 Mb, 499–500 Mb, 511–513 Mb, and 520–524 Mb regions of chromosome 3B (https://urgi.versailles.inra.fr/gb2/gbrowse/wheat_annot_3B/) (Table 3). The newly developed SSR markers were first screened through lm3 and Jingdong8, and then these polymorphic markers were applied to a subset population including 24 segregating F_7:8_ individuals. Moreover, six markers (*SSR3B400*.*4*, *SSR3B450*.*37*, *SSR3B492*.*6*, *SSR3B499*.*24*, *SSR3B511*.*2* and *SSR3B520*.*6*) were closely linked with *lm3*, and these markers were further assayed using the entire F_7:8_ segregating population. The linkage analysis was performed using the genotypes of SSR markers and the phenotypic data of the entire F_7:8_ population, and the *lm3* locus was flanked by *SSR3B450*.*37* and *SSR3B492*.*6* at genetic distances of 2.07 cM and 0.95 cM, respectively (Fig 6B). To delimit the *lm3* locus to a narrow region, the 482–484 Mb, 487–489 Mb, and 490–492 Mb regions of chromosome 3B were subjected to SSR development, and SSR markers with polymorphisms between two parents were monomorphic between individuals with or without lesion symptoms, failing to map in the genetic linkage map. Thus, *lm3* was mapped to a 40 Mb region on chromosome 3B, flanked by *SSR3B450*.*37* and *SSR3B492*.*6*.

**Table 3:** Details of SSR markers used to map the lm3 locus.

SSR	Forward primer (5'-3')	Reverse primer (5'-3')	Sequence ID or Position in the 3B genome (Mb)
BARC1140	ACTGTGTGGGTTGTCTGAGGTCATT	GCGTTATGAAATCTTTGTTCCGTAATA	BV211508
BARC203	GCGAACATGTATCCAAGTCACTAACC	GCGCCACATTCAAACATAAGGTCATT	BV211642
BARC268	GCGATTCCTTTGTTCCTTCCCCATAC	GCAGCATGTCTAGCCAACTTGTCGTG	BV211841
WMC653	AGTGTTTTAGGGGTGGAAGGGA	CGGAACCCTAAACCCTAGTCG	NA
GWM376	GGGCTAGAAAACAGGAAGGC	TCTCCCGGAGGGTAGGAG	NA
GWM644	GTGGGTCAAGGCCAAGG	AGGAGTAGCGTGAGGGGC	NA
CFP2126	CGCTTGATCCCTCTCGGTACATC	AGGCCCGGAACATGGCAG	DX380955
SSR3B400.4	GAGATGAAGATCCGGTGAGGT	CATACCCATTCAACTCAGATTCC	400.280
SSR3B450.37	CAATCGGTAGATCTGACTAATACA	TGACTGAATAATATCTCACACACA	451.797
SSR3B492.6	ATATCAGAAACGTTTTCTCCTTAC	AGCACTTGTTTCTTTGTTTTCT	492.344
SSR3B499.24	AAATGATCGTAAGATTACCTCG	GTTGCATCACTTTTTACTTTACAT	499.848
SSR3B511.2	ATATATGGTGCATTATGAACTTGT	AGGTATGCGATGTAATACTCTTTA	511.807
SSR3B520.6	GTCAACTTTTTAACAACACACTTC	GTATTTTCCCCTATAAACATTTTG	521.579

## Discussion

### A new type of LM in wheat

The *lm3* mutant is a novel LMM of wheat, characterized by the spontaneous cell death phenotype typical to previously reported LMMs (Fig 1). This mutant likely originated from a natural mutation in the field, expressed as small, discrete white lesions on the leaf blades during the early stages of development, and expanding into large whitish-brown necrotic spots on the leaf blade and leaf sheaths in late stages of development, particularly after the anthesis stage, during which the time and intensity of solar illumination increased markedly in the field. On a specific leaf blade, lesions originated from the leaf tips and subsequently extended to the entire leaf blade. The stages and patterns of lesion development in the *lm3* mutant differed from those reported in previous studies. The development of lesions in the C591 (M8) mutant arising from N-nitroso methyl urethane treatment is only observed beginning at the booting stage, progressing from the lowest leaf sheath and leaf toward the top; the appearance of spots is random, and the spotting intensity varies on a particular leaf blade and leaf sheath [64]. The M66 lesion mutant obtained through X-ray mutagenesis expressed necrotic spots on the leaves at the young seedling stage with the main shoot and two or three tillers and continued to develop up to seed setting [65].The lesions in the HLP mutant, induced through ethyl methane sulfonate (EMS), only became evident on the leaf tips at the fifth or sixth leaf stage and randomly spread along the leaf blades, the leaf sheathes and flowering tissues; however these lesions were small (1–2 mm) and white in color [37]. The spotted-leaf mutant LF2010A was also obtained through EMS treatment; this mutant initially shows bright yellow spots on its primary leaves at the three-leaf stage, which subsequently spread to the leaves and leaf sheaths of the entire plant, including spike tissues [14]. The natural LM mutant in Ning7840 spontaneously exhibited small, discrete, yellowish spots only on the leaf blades around the heading stage [12]. Different from previous lesion mutants, a novel lesion-mimic phenotype, appearing in a segregating population of the common wheat cross Yanzhan1/Zaosui30, was controlled through the interaction of two recessive genes. The lesion-mimic phenotype became most obvious at the booting stage, progressively developing from the lower leaves to the flag leaf and characterizing discrete brown lesions on the leaf blades, leaf sheathes and flowering tissues [13]. Moreover, most of those LMMs are controlled by recessive genes [12–14, 39], but only a few mutations are regulated by dominant genes [64]. In the present study, it could be concluded that the lm3 mutant had newly arisen because its LM phenotype was controlled by a partially dominant gene. In the heterozygous F_1_ individual, the lesions were less abundant and smaller than those in the homozygous *lm3* mutant, and the chlorophyll content also decreased to values observed between those of the two parents (lm3 and 3–1) (Fig 1A and 1B).

### Chromosome location of *lm3*

Many LMMs have been identified in higher plants, particularly in Arabidopsis, rice and maize, and a number of genes conferring the corresponding phenotype have been mapped onto chromosomes, and even cloned with the availability of the genome sequences. In hexaploid wheat, several LMMs have been characterized using a variety of methods [12–14, 37, 64, 65], whereas few genes have been mapped and cloned, reflecting the genome complexity and insufficiency of the genome sequences. The LM in Ning7840 was mapped to chromosome 1BL 15 cM away from the closest markers *Xwmc85*.*1* and *Xwmc264*.*1* [12]. MNR220 is located on chromosome 2BS flanked by XBE497494 (9.3 cM) and Xbarc183 (12.6 cM), and is responsible for the pathogen-induced necrotic spots observed in a broad-spectrum disease resistant mutant [66]. Two interactive loci (*lm1* and *lm2*) resulting in the LM phenotype in a segregating population of the common wheat cross Yanzhan1/Zaosui30 were mapped to chromosomes 3BS flanked between *Xwmc674* and *Xbarc133*/*Xbarc147* and 4BL between *Xgwm513* and *Xksum154*, respectively. In the present study, *lm3* was mapped to chromosome 3BL, close to the centromere region, different from the *lm1* locus on chromosome 3BS [13]. Taken together, among the mapped loci/genes for the LM phenotype, most loci were mapped on B genomes, suggesting that these B genomes harbor more necrosis-related genes or are prone to mutation.

Because the complete 3B genome sequence of Chinese Spring is available [67], fine mapping and map-based cloning of the *lm3* locus was attempted using the derived F_7:8_ segregating population obtained from the lm3/Jingdong8 cross. Several dozens of SSR markers were designed within the 400 to 520 Mb genome regions of chromosome 3B. These SSR markers were screened with lm3, Jingdong8 and a subset of the derived F_7:8_ segregating population, and only a few markers could be anchored to the *lm3* locus map of chromosome 3B after genotyping 5376 individuals. This result revealed the flanking of the *lm3* locus within *SSR3B450*.*37* and *SSR3B492*.*6*, having the genetic distances of 2.07 cM and 0.95 cM, respectively (Fig 6B). To fine map the *lm3* locus, additional SSR markers were designed in the regions of 482 to 490 Mb, but these markers failed to reveal polymorphisms between individuals with or without the LM symptoms even though several markers were polymorphic between the two parents of the initial population. In the present study, the strategy of the derived segregating population was explored [68], the heterozygous individuals were preserved and self-pollinated in each segregating generation from the F_2_ generation, and a F_7:8_ segregating population comprising 5,376 individuals was developed from twenty self-pollinated F_7_ individuals. Selection based on the phenotypic data might lead to the recombination between the *lm3* locus, and these recombinants were selected and propagated, causing monomorphisms between individuals with or without LM symptoms. Moreover, the *lm3* locus in 3BL was located near the centromere region, where low polymorphisms were generally detected among the different lines. Several F_2_ populations were currently being developed to solve these problems.

### Light requirements for the occurrence of LMs

The formation of plant LMs is correlated with the disturbance of the regulation of metabolic pathways through related gene mutations, and is also associated with environmental factors, including light, temperature, humidity and nutrition [24, 56, 69, 70]. The size of the rice *spl1*, *spl3* and *spl4* lesions increased with light intensity and elevated temperature [24]. Variable lesion densities were observed on the rice *spl7* mutant at different temperatures and ultraviolet solar irradiation, with the density decreasing at low temperatures [15]. The *spl30(t)* lesions were induced through light, including fluorescent light, and enhanced by higher temperatures [22], whereas the lesions of the rice *Oslsd1* mutant were induced at a lower temperature and under shorter duration of daylight [1]. Moreover, the expression of lesion spots in *spl30* mutants is sensitive to light and humidity [61]. In the present study, no visible lesions were detected under the 0 -h photoperiod, but lesions appeared under illumination, indicating that the mutant was light dependent, similar to the LF2010A mutant [14] and the LM lines derived from the Yanzhan1/Zaosui30 cross [13]. In addition, the size and number of lesions significantly increased under the conditions of higher light intensity and longer photoperiods (Fig 3). These data suggested that both light intensity and photoperiod play essential roles in lesion formation. However, additional studies on responses to the specific spectrum of light and the mechanisms of the light-dependent lesion formation in this mutant should be considered.

### Expression of defense-related genes

Lesion mimic mutants are interesting genetic materials for dissecting pathways of disease resistance and PCD. Thus far, 37 of the 49 identified spotted-leaf mutants of rice have been reported to show enhanced resistance to at least one pathogen [22]. Although the detailed underlying mechanism remains poorly understood, a wide range of defense genes have been implicated in an intricate network of defense signaling pathways associated with resistance to microbial pathogens [71, 72]. The elevated expression of these defense genes accompanied the pathogen inoculation, and the over-expression of a range of genes has led to improved disease resistance [73, 74]. In the present study, we speculated that the initiation of lesions in the *lm3* mutant might also be associated with the activation of defense genes, particularly pathogen-related genes linked to resistance to powdery mildew. To examine this possibility, we analyzed the expression of a panel of wheat defense genes including representatives of the *WCI* and *PR* gene families [43]. Five *PR* and two *WCI* genes were significantly up-regulated during the initiation of lesion formation in the *lm3* mutant, and the elevated fold-changes of these genes decreased once the lesions were fully developed. The *WCI* genes are a specific set of genes induced through benzo(1,2,3)thiadiazole-7-carbothionic acid S-methyl ester (BTH), and this induction increased protection against the wheat powdery mildew fungus *Blumeria graminis* f. sp. *tritici* [75]. The expression of *WCI2* and *WCI3* in the *lm3* mutant was induced through the initiation of lesion formation, and the expression of these genes remained at a higher level after lesion formation, suggesting that both genes are involved in the HR of PCD and ROS accumulation. Two other *PR* genes, *PR1* and *TaGLP2a* showed stable up-regulation during lesion formation in the *lm3* mutant, indicating that the potential constitutive activation of a defense signaling pathway was preserved after lesion initiation. *TaGLP2a* is strongly induced by *Fusarium pseudograminearum*, methyl jasmonate [43], and *Blumeria graminis* [76], and the encoded protein displays functions involved in resistance, as transient overexpression in wheat cells enhances resistance against *B*. *graminis*, whereas transient silencing through RNA interference reduces resistance [77]. *PR1*, which is considered a defense marker in plant–pathogen interactions, is induced by a wide-range of microbial pathogens, environmental stresses and chemicals [43, 78] and over-expression of this gene confers improved resistance to the powdery mildew fungus *Peronosclerospora sorghi* in maize [59]. In the *lm3* mutant, the up-regulated expression of these defense genes suggested that *lm3* acts as a positive regulator in the plant resistance signaling pathway. In contrast, the transcript abundance of *PWIR2*, a thaumatin-like protein induced in plant defense during pathogen attack [63], remained unchanged during lesion formation. However, the expression of this gene was two-fold higher in root samples from the *lm3* mutant even though no lesions were detected in the roots. This phenomenon was also observed in *WCI3* and *TaGLP2a* (Fig 5), suggesting that root tissues responded to lesion formation in the shoots. Therefore, further cloning of the *lm3* gene will provide new insights into the molecular mechanisms of cell death and disease resistance signaling in plants.

## References

[1] Wang J, Ye BQ, Yin JJ, Yuan C, Zhou XG, Li WT, et al Characterization and fine mapping of a light-dependent *leaf lesion mimic mutant 1* in rice. Plant Physiol Biochem. 2015;97:44–51. 10.1016/j.plaphy.2015.09.001 . 26410574

[2] Negishi K, Williams DM, Inoue Y, Moriyama K, Brown DM, Hayatsu H. The mechanism of mutation induction by a hydrogen bond ambivalent, bicyclic N^4^-oxy-2'-deoxycytidine in *Escherichia coli*. Nucleic Acids Res. 1997;25(8):1548–52. 9092660 10.1093/nar/25.8.1548 PMC146628

[3] Lam E, Kato N, Lawton M. Programmed cell death, mitochondria and the plant hypersensitive response. Nature. 2001;411(6839):848–53. 10.1038/35081184 . 11459068

[4] Dangl JL, Dietrich RA, Richberg MH. Death don't have no mercy: Cell death programs in plant-microbe interactions. Plant Cell. 1996;8(10):1793–807. . 12239362 10.1105/tpc.8.10.1793 PMC161315

[5] Muthamilarasan M, Prasad M. Plant innate immunity: An updated insight into defense mechanism. J Biosci. 2013;38(2):433–49. 10.1007/s12038-013-9302-2 . 23660678

[6] Greenberg JT. Programmed cell death In plant-pathogen interactions. Annu Rev Plant Biol. 1997;48:525–45. 10.1146/annurev.arplant.48.1.525 . 15012273

[7] Yang Ml, Wardzala E, Johal GS, Gray J. The wound-inducible *Lls1* gene from maize is an orthologue of the *Arabidopsis Acd1* gene, and the LLS1 protein is present in non-photosynthetic tissues. Plant Mol Bio. 2004;54:175–91. 15159621 10.1023/B:PLAN.0000028789.51807.6a

[8] Penning BW, Johal GS, McMullen MD. A major suppressor of cell death, *slm1*, modifies the expression of the maize (*Zea mays* L.) lesion mimic mutation *les23*. Genome. 2004;47(5):961–9. 10.1139/g04-046 . 15499410

[9] Zeng LR, Qu S, Bordeos A, Yang C, Baraoidan M, Yan H, et al *Spotted leaf11*, a negative regulator of plant cell death and defense, encodes a U-box/armadillo repeat protein endowed with E3 ubiquitin ligase activity. Plant Cell. 2004;16(10):2795–808. 10.1105/tpc.104.025171 15377756 PMC520972

[10] Chen XF, Hao L, Pan JW, Zheng XX, Jiang GH, Jin Y, et al *SPL5*, a cell death and defense-related gene, encodes a putative splicing factor 3b subunit 3 (SF3b3) in rice. Mol Breeding. 2012;30(2):939–49. 10.1007/s11032-011-9677-4 .

[11] Ge CW, E ZG, Pan JJ, Jiang H, Zhang XQ, Zeng DL, et al Map-based cloning of a spotted-leaf mutant gene *OsSL5* in *Japonica* rice. Plant Growth Regul. 2014;75(3):595–603. 10.1007/s10725-014-9962-4

[12] Li T, Bai GH. Lesion mimic associates with adult plant resistance to leaf rust infection in wheat. Theor Appl Genet. 2009;119(1):13–21. 10.1007/s00122-009-1012-7 . 19330313

[13] Yao Q, Zhou RH, Fu TH, Wu WR, Zhu ZD, Li AL, et al Characterization and mapping of complementary lesion-mimic genes *lm1* and *lm2* in common wheat. Theor Appl Genet. 2009;119(6):1005–12. 10.1007/s00122-009-1104-4 . 19621213

[14] Du LF, Li MF, Liu LX, Wang CJ, Liu Y, Xu XT, et al Physiological characteristics and genetic analysis on a spotted-leaf wheat derived from chemical mutation. Acta Agronomica Sinica. 2014;40(6):1020–6. 10.3724/sp.j.1006.2014.01020

[15] Yamanouchi U, Yano M, Lin HX, Ashikari M, Yamada K. A rice spotted leaf gene, *SpI7*, encodes a heat stress transcription factor protein. Proc Natl Acad Sci U S A. 2002;99(11):7530–5. 10.1073/pnas.112209199 . 12032317 PMC124274

[16] Sun CH, Liu LC, Tang JY, Lin AH, Zhang FT, Fang J, et al *RLIN1*, encoding a putative coproporphyrinogen III oxidase, is involved in lesion initiation in rice. J Genet and Genomics. 2011;38(1):29–37. 10.1016/j.jcg.2010.12.001 . 21338950

[17] Fekih R, Tamiru M, Kanzaki H, Abe A, Yoshida K, Kanzaki E, et al The rice (*Oryza sativa* L.) *LESION MIMIC RESEMBLING*, which encodes an AAA-type ATPase, is implicated in defense response. Mol Genet and Genomics. 2015;290(2):611–22. 10.1007/s00438-014-0944-z . 25367283

[18] Qiao YL, Jiang WZ, Lee J, Park B, Choi MS, Piao RH, et al *SPL28* encodes a clathrin-associated adaptor protein complex 1, medium subunit micro 1 (AP1M1) and is responsible for spotted leaf and early senescence in rice (*Oryza sativa*). New Phytol. 2010;185(1):258–74. 10.1111/j.1469-8137.2009.03047.x . 19825016

[19] Wang SH, Lim JH, Kim SS, Cho SH, Yoo SC, Koh HJ, et al Mutation of *SPOTTED LEAF3* (*SPL3*) impairs abscisic acid-responsive signalling and delays leaf senescence in rice. J Exp Bot. 2015 10.1093/jxb/erv401.26276867 PMC4765782 26276867

[20] Wang ZH, Wang Y, Hong X, Hu DH, Liu CX, Yang J, et al Functional inactivation of UDP-*N*-acetylglucosamine pyrophosphorylase 1 (UAP1) induces early leaf senescence and defence responses in rice. J Exp Bot. 2015;66(3):973–87. 10.1093/jxb/eru456 25399020 PMC4321554

[21] Li Z, Zhang YX, Liu L, Liu QN, Bi ZZ, Yu N, et al Fine mapping of the lesion mimic and early senescence 1 (*lmes1*) in rice (*Oryza sativa*). Plant Physiol Biochem. 2014;80:300–7. 10.1016/j.plaphy.2014.03.031 . 24832615

[22] Huang QN, Shi YF, Yang Y, Feng BH, Wei YL, Chen J, et al Characterization and genetic analysis of a light- and temperature-sensitive spotted-leaf mutant in rice. J Integr Plant Biol. 2011;53(8):671–81. 10.1111/j.1744-7909.2011.01056.x . 21605341

[23] Kojo K, Yaeno T, Kusumi K, Matsumura H, Fujisawa S, Terauchi R, et al Regulatory mechanisms of ROI generation are affected by rice *spl* mutations. Plant Cell physiol. 2006;47(8):1035–44. 10.1093/pcp/pcj074 . 16816407

[24] Matin MN, Saief SA, Rahman MM, Lee DH, Kang H, Lee DS, et al Comparative phenotypic and physiological characteristics of spotted leaf 6 (*spl6*) and brown leaf spot2 (*bl2*) lesion mimic mutants (LMM) in rice. Mol Cells. 2010;30(6):533–43. 10.1007/s10059-010-0151-7 . 21110131

[25] Ishikawa A, Tanaka H, Nakai M, Asahi T. Deletion of a chaperonin 60β gene leads to cell death in the Arabidopsis lesion initiation 1 mutant. Plant Cell Physiol. 2003;44(3):255–61. . 12668771 10.1093/pcp/pcg031

[26] Landoni M, De Francesco A, Bellatti S, Delledonne M, Ferrarini A, Venturini L, et al A mutation in the *FZL* gene of *Arabidopsis* causing alteration in chloroplast morphology results in a lesion mimic phenotype. J Exp Bot. 2013;64(14):4313–28. 10.1093/jxb/ert237 23963675 PMC3808314

[27] Quesada V, Sarmiento-Manus R, Gonzalez-Bayon R, Hricova A, Ponce MR, Micol JL. *PORPHOBILINOGEN DEAMINASE* deficiency alters vegetative and reproductive development and causes lesions in Arabidopsis. PLoS One. 2013;8(1):e53378 10.1371/journal.pone.0053378 23308205 PMC3540089

[28] Guo CY, Wu GH, Xing J, Li WQ, Tang DZ, Cui BM. A mutation in a coproporphyrinogen III oxidase gene confers growth inhibition, enhanced powdery mildew resistance and powdery mildew-induced cell death in Arabidopsis. Plant Cell Rep. 2013;32(5):687–702. 10.1007/s00299-013-1403-8 . 23462936

[29] Ishikawa A, Okamoto H, Iwasaki Y, Asahi T. A deficiency of coproporphyrinogen III oxidase causes lesion formation in Arabidopsis. Plant J. 2001;27(2):89–99. . 11489187 10.1046/j.1365-313x.2001.01058.x

[30] Jambunathan N, Siani JM, McNellis TW. A humidity-sensitive Arabidopsis copine mutant exhibits precocious cell death and increased disease resistance. Plant Cell. 2001;13(10):2225–40. 11595798 10.1105/tpc.010226 PMC139155

[31] Shirano Y, Kachroo P, Shah J, Klessig DF. A gain-of-function mutation in an Arabidopsis Toll interleukin1 receptor-nucleotide binding site-leucine-rich repeat type R gene triggers defense responses and results in enhanced disease resistance. Plant Cell. 2002;14(12):3149–62. 10.1105/tpc.005348 12468733 PMC151208

[32] Balague C, Lin B, Alcon C, Flottes G, Malmstrom S, Kohler C, et al HLM1, an essential signaling component in the hypersensitive response, is a member of the cyclic nucleotide-gated channel ion channel family. Plant Cell. 2003;15(2):365–79. 10.1105/tpc.006999 12566578 PMC141207

[33] Zhang CJ, Ouyang B, Yang CX, Zhang XH, Liu H, Zhang YY, et al Reducing AsA leads to leaf lesion and defence response in knock-down of the AsA biosynthetic enzyme GDP-D-mannose pyrophosphorylase gene in tomato plant. PLoS One. 2013;8(4). 10.1371/journal.pone.0061987.g001 PMC3633959 23626761

[34] Zhang Y, Zhang YH, Qiu DW, Zeng HM, Guo LH, Yang XF. BcGs1, a glycoprotein from *Botrytis cinerea*, elicits defence response and improves disease resistance in host plants. Biochem Biophys Res Commun. 2015;457(4):627–34. 10.1016/j.bbrc.2015.01.038 . 25613865

[35] Kumar V, Parkhi V, Joshi SG, Christensen S, Jayaprakasha GK, Patil BS, et al A novel, conditional, lesion mimic phenotype in cotton cotyledons due to the expression of an endochitinase gene from *Trichoderma virens*. Plant Sci. 2012;183:86–95. 10.1016/j.plantsci.2011.11.005 . 22195581

[36] Sun LQ, Zhu LF, Xu L, Yuan DJ, Min L, Zhang XL. Cotton cytochrome P450 CYP82D regulates systemic cell death by modulating the octadecanoid pathway. Nat Commun. 2014;5:5372 10.1038/ncomms6372 25371113 PMC4241986

[37] Kamlofski CA, Antonelli E, Bender C, Jaskelioff M, Danna CH, Ugalde R, et al A lesion-mimic mutant of wheat with enhanced resistance to leaf rust. Plant Pathol. 2007;56(1):46–54. 10.1111/j.1365-3059.2006.01454.x .

[38] Kinane JT, Jones PW. Isolation of wheat mutants with increased resistance to powdery mildew from small induced variant populations. Euphytica. 2001;117(3):251–60. 10.1023/A:1026527010901 .

[39] Luo PG, Ren ZL. Wheat leaf chlorosis controlled by a single recessive gene. Journal Of Plant Physiology and Molecular Biology. 2006;32(3):330–8. . 16775402

[40] Arnon DI, Whatley FR. Factors influencing oxygen production by illuminated chloroplast fragments. Arch Biochem. 1949;23(1):141–56. . 18135772

[41] Yin Z, Chen J, Zeng L, Goh M, Leung H, Khush GS, et al Characterizing rice lesion mimic mutants and identifying a mutant with broad-spectrum resistance to rice blast and bacterial blight. Mol Plant Microbe Interact. 2000;13(8):869–76. 10.1094/MPMI.2000.13.8.869 . 10939258

[42] Thordal-Christensen H, Zhang ZG, Wei YD, Collinge DB. Subcellular localization of H_2_O_2_ in plants. H_2_O_2_ accumulation in papillae and hypersensitive response during the barley-powdery mildew interaction. Plant J. 1997;11(6):1187–94. 10.1046/j.1365-313X.1997.11061187.x .

[43] Desmond OJ, Edgar CI, Manners JM, Maclean DJ, Schenk PM, Kazan K. Methyl jasmonate induced gene expression in wheat delays symptom development by the crown rot pathogen *Fusarium pseudograminearum*. Physiol Mol Plant P. 2005;67(3–5):171–9. 10.1016/j.pmpp.2005.12.007

[44] Sambrook J, Russell DW. Purification of RNA from cells and tissues by Acid phenol-guanidinium thiocyanate-chloroform extraction. CSH Protoc. 2006;2006(1). 10.1101/pdb.prot4045 . 22485463

[45] Paolacci AR, Tanzarella OA, Porceddu E, Ciaffi M. Identification and validation of reference genes for quantitative RT-PCR normalization in wheat. BMC Mol Biol. 2009;10:11 10.1186/1471-2199-10-11 19232096 PMC2667184

[46] Saghai-Maroof MA, Soliman KM, Jorgensen RA, Allard RW. Ribosomal DNA spacer-length polymorphisms in barley: mendelian inheritance, chromosomal location, and population dynamics. Proc Natl Acad Sci U S A. 1984;81(24):8014–8. 6096873 10.1073/pnas.81.24.8014 PMC392284

[47] Michelmore RW, Paran I, Kesseli RV. Identification of markers linked to disease-resistance genes by bulked segregant analysis: a rapid method to detect markers in specific genomic regions by using segregating populations. Proc Natl Acad Sci U S A. 1991;88(21):9828–32. 1682921 10.1073/pnas.88.21.9828 PMC52814

[48] Song QJ, Shi JR, Singh S, Fickus EW, Costa JM, Lewis J, et al Development and mapping of microsatellite (SSR) markers in wheat. Theor Appl Genet. 2005;110(3):550–60. 10.1007/s00122-004-1871-x . 15655666

[49] Guyomarc'h H, Sourdille P, Edwards J, Bernard M. Studies of the transferability of microsatellites derived from *Triticum tauschii* to hexaploid wheat and to diploid related species using amplification, hybridization and sequence comparisons. Theor Appl Genet. 2002;105(5):736–44. 10.1007/s00122-002-0963-8 . 12582487

[50] Sourdille P, Cadalen T, Guyomarc'h H, Snape JW, Perretant MR, Charmet G, et al An update of the Courtot x Chinese Spring intervarietal molecular marker linkage map for the QTL detection of agronomic traits in wheat. Theor Appl Genet. 2003;106(3):530–8. 10.1007/s00122-002-1044-8 . 12589554

[51] Bassam BJ, Caetano-Anolles G, Gresshoff PM. Fast and sensitive silver staining of DNA in polyacrylamide gels. Anal Biochem. 1991;196(1):80–3. . 1716076 10.1016/0003-2697(91)90120-i

[52] da Maia LC, Palmieri DA, de Souza VQ, Kopp MM, de Carvalho FI, Costa de Oliveira A. SSR locator: tool for simple sequence repeat discovery integrated with primer design and PCR simulation. Int J Plant Genomics. 2008;2008:412696 10.1155/2008/412696 18670612 PMC2486402

[53] Lander ES, Green P, Abrahamson J, Barlow A, Daly MJ, Lincoln SE, et al MAPMAKER: an interactive computer package for constructing primary genetic linkage maps of experimental and natural populations. Genomics. 1987;1(2):174–81. . 3692487 10.1016/0888-7543(87)90010-3

[54] Kosambi DD. The estimation of map distance from recombination values. Ann Hum Genet. 1943;12(1):172–5. 10.1111/j.1469-1809.1943.tb02321.x

[55] Voorrips RE. MapChart: software for the graphical presentation of linkage maps and QTLs. J Hered. 2002;93(1):77–8. . 12011185 10.1093/jhered/93.1.77

[56] Arase S, Fujita K, Uehara T, Honda Y, Isota J. Light-enhanced resistance to *Magnaporthe grisea* infection in the rice *Sekiguchi lesion* mutants. J Phytopathol. 2000;148(4):197–203.

[57] Gray J, Janick-Buckner D, Buckner B, Close PS, Johal GS. Light-dependent death of maize lls1 cells is mediated by mature chloroplasts. Plant Physiol. 2002;130(4):1894–907. 10.1104/pp.008441 12481072 PMC166700

[58] Hu G, Yalpani N, Briggs SP, Johal GS. A porphyrin pathway impairment is responsible for the phenotype of a dominant disease lesion mimic mutant of maize. Plant Cell. 1998;10(7):1095–105. 9668130 10.1105/tpc.10.7.1095 PMC144048

[59] Morris SW, Vernooij B, Titatarn S, Starrett M, Thomas S, Wiltse CC, et al Induced resistance responses in maize. Mol Plant Microbe Interact. 1998;11(7):643–58. 10.1094/MPMI.1998.11.7.643 . 9650297

[60] Dietrich RA, Delaney TP, Uknes SJ, Ward ER, Ryals JA, Dangl JL. Arabidopsis mutants simulating disease resistance response. Cell. 1994;77(4):565–77. . 8187176 10.1016/0092-8674(94)90218-6

[61] Xu X, Zhang LL, Liu BM, Ye YF, Wu YJ. Characterization and mapping of a spotted leaf mutant in rice (*Oryza sativa*). Genet Mol Biol. 2014;37(2):406–13. 25071406 10.1590/s1415-47572014005000001 PMC4094620

[62] Zhang Z, Lenk A, Andersson MX, Gjetting T, Pedersen C, Nielsen ME, et al A lesion-mimic syntaxin double mutant in Arabidopsis reveals novel complexity of pathogen defense signaling. Mol Plant. 2008;1(3):510–27. 10.1093/mp/ssn011 . 19825557

[63] Desmond OJ, Manners JM, Stephens AE, Maclean DJ, Schenk PM, Gardiner DM, et al The *Fusarium* mycotoxin deoxynivalenol elicits hydrogen peroxide production, programmed cell death and defence responses in wheat. Mol Plant Pathol. 2008;9(4):435–45. 10.1111/j.1364-3703.2008.00475.x . 18705859 PMC6640518

[64] Nair SK, Tomar SMS. Genetical and anatomical analyses of a leaf flecking mutant in *Triticum aestivum* L. Euphytica. 2001;121(1):53–8.

[65] Boyd LA, Smith PH, Wilson AH, Minchin PN. Mutations in wheat showing altered field resistance to yellow and brown rust. Genome. 2002;45(6):1035–40. . 12502247 10.1139/g02-072

[66] Campbell J, Zhang HT, Giroux MJ, Feiz L, Jin Y, Wang MN, et al A mutagenesis-derived broad-spectrum disease resistance locus in wheat. Theor Appl Genet. 2012;125(2):391–404. 10.1007/s00122-012-1841-7 22446929 PMC3374107

[67] Choulet F, Alberti A, Theil S, Glover N, Barbe V, Daron J, et al Structural and functional partitioning of bread wheat chromosome 3B. Science. 2014;345(6194):1249721 10.1126/science.1249721 . 25035497

[68] Yang Q, Zhang DF, Xu ML. A sequential quantitative trait locus fine-mapping strategy using recombinant-derived progeny. J Integr Plant Biol. 2012;54(4):228–37. 10.1111/j.1744-7909.2012.01108.x . 22348858

[69] Fuse T, Iba K, Satoh H, Nishimura M. Characterization of a rice mutant having an increased susceptibility to light stress at high temperature. Physiol Plantarum. 1993;89(4):799–804. 10.1111/j.1399-3054.1993.tb05287.x

[70] Lorrain S, Vailleau F, Balague C, Roby D. Lesion mimic mutants: keys for deciphering cell death and defense pathways in plants? Trends Plant Sci. 2003;8(6):263–71. 10.1016/S1360-1385(03)00108-0 . 12818660

[71] Mizobuchi R, Hirabayashi H, Kaji R, Nishizawa Y, Yoshimura A, Satoh H, et al Isolation and characterization of rice lesion-mimic mutants with enhanced resistance to rice blast and bacterial blight. Plant Sci. 2002;163(2):345–53. 10.1016/S0168-9452(02)00134-6 .

[72] Campbell MA, Ronald PC. Characterization of four rice mutants with alterations in the defence response pathway. Mol Plant Pathol. 2005;6(1):11–21. 10.1111/j.1364-3703.2004.00256.x . 20565634

[73] Takahashi A, Kawasaki T, Henmi K, Shii K, Kodama O, Satoh H, et al Lesion mimic mutants of rice with alterations in early signaling events of defense. Plant J. 1999;17(5):535–45. 10.1046/j.1365-313X.1999.00405.x 10205906

[74] Mackintosh CA, Lewis J, Radmer LE, Shin S, Heinen SJ, Smith LA, et al Overexpression of defense response genes in transgenic wheat enhances resistance to Fusarium head blight. Plant Cell Rep. 2007;26(4):479–88. 10.1007/s00299-006-0265-8 17103001 PMC1824786

[75] Gorlach J, Volrath S, Knauf-Beiter G, Hengy G, Beckhove U, Kogel KH, et al Benzothiadiazole, a novel class of inducers of systemic acquired resistance, activates gene expression and disease resistance in wheat. Plant Cell. 1996;8(4):629–43. 10.1105/tpc.8.4.629 8624439 PMC161125

[76] Schweizer P, Christoffel A, Dudler R. Transient expression of members of the germin-like gene family in epidermal cells of wheat confers disease resistance. Plant J. 1999;20(5):541–52. 10.1046/j.1365-313X.1999.00624.x 10652126

[77] Christensen AB, Thordal-Christensen H, Zimmermann G, Gjetting T, Lyngkjær MF, Dudler R, et al The germinlike protein GLP4 exhibits superoxide dismutase activity and is an important component of quantitative resistance in wheat and barley. Mol Plant Microbe Interact. 2004;17(1):109–17. 14714874 10.1094/MPMI.2004.17.1.109

[78] Mitsuhara I, Iwai T, Seo S, Yanagawa Y, Kawahigasi H, Hirose S, et al Characteristic expression of twelve rice PR1 family genes in response to pathogen infection, wounding, and defense-related signal compounds (121/180). Mol Genet Genomics. 2008;279(4):415–27. 10.1007/s00438-008-0322-9 18247056 PMC2270915

